# Impact of COVID-19 on Hospital Admissions and Healthcare Quality Indicators in Non-COVID Patients: A Retrospective Study of the First COVID-19 Year in a University Hospital in Spain

**DOI:** 10.3390/jcm11071752

**Published:** 2022-03-22

**Authors:** Laia Domingo, Mercè Comas, Anna Jansana, Javier Louro, Helena Tizón-Marcos, Maria Lourdes Cos, Jaume Roquer, Juan José Chillarón, Isabel Cirera, Sergi Pascual-Guàrdia, Maria Sala, Xavier Castells

**Affiliations:** 1 Department of Epidemiology and Evaluation, Hospital del Mar-IMIM, 08003 Barcelona, Spain; mcomas@psmar.cat (M.C.); jansanariera.anna@gmail.com (A.J.); jlouro@imim.es (J.L.); msalaserra@psmar.cat (M.S.); xcastells@psmar.cat (X.C.); 2 Research Network on Health Services in Chronic Diseases (REDISSEC), RICAPPS, Instituto de Salud Carlos III, 28029 Madrid, Spain; 3 Department of Cardiology, Hospital del Mar-IMIM, 08003 Barcelona, Spain; htizon@psmar.cat; 4 Centro de Investigación Biomédica en Red Enfermedades Cardiovasculares (CIBERCV), Instituto de Salud Carlos III, 28029 Madrid, Spain; 5 Department of Internal Medicine, Hospital del Mar-IMIM, Universitat Autònoma de Barcelona, 08003 Barcelona, Spain; mcos@psmar.cat; 6 Neurology Department, Hospital del Mar-IMIM, 08003 Barcelona, Spain; jroquer@psmar.cat; 7 Department of Medicine and Life Sciences (MELIS), Universitat Pompeu Fabra, 08003 Barcelona, Spain; icirera@psmar.cat (I.C.); spascual@psmar.cat (S.P.-G.); 8 Department of Endocrinology and Nutrition, Hospital del Mar-IMIM, 08003 Barcelona, Spain; jchillaron@psmar.cat; 9 Emergency Department, Hospital del Mar-IMIM, 08003 Barcelona, Spain; 10 Pneumology Department, Hospital del Mar-IMIM, 08003 Barcelona, Spain; 11 Centro de Investigación Biomédica en Red Enfermedades Respiratorias (CIBERES), Instituto de Salud Carlos III, 28029 Madrid, Spain

**Keywords:** COVID-19, impact, healthcare quality, lockdown, hospital admissions, complications, in-hospital mortality

## Abstract

Few studies have assessed the impact of the COVID-19 pandemic on non-COVID diseases and healthcare quality. We aimed to evaluate changes in rates of hospitalisations, complications, in-hospital mortality, and readmissions among patients with non-COVID diseases during a one-year period after the pandemic onset. From March 2018 to February 2021 a retrospective observational study of hospital admissions in a university hospital in Spain was conducted. Non-COVID hospitalisations admitted through the emergency department were compared between the pre-COVID period (*n* = 28,622) and the COVID period (*n* = 11,904). We assessed rate ratios (RaR), comparing the weekly number of admissions and risk ratios (RR) to examine rates of complications, in-hospital mortality, readmissions, and severity. Statistical significance was set at *p* < 0.05. The weekly admission rate dropped by 20.8% during the complete lockdown. We observed significant reductions in admissions related to diseases of the respiratory system and circulatory system. Admissions for endocrine and metabolic diseases increased. The complication rates increased (RR = 1.21, 95% CI: 1.05;1.4), while in-hospital mortality rates held steady during the COVID period (RR = 1.09, 95% CI: 0.98;1.2). Hospital efforts to maintain quality and safety standards despite disruptions translated into a moderate increase in complications but not in in-hospital mortality. Reduced hospitalisations for conditions requiring timely treatment may have significant public health consequences.

## 1. Introduction

The World Health Organisation declared SARS-CoV-2 and its resulting infection, COVID-19 (coronavirus infectious disease 2019), a global pandemic on 11 March 2020 when the number of infections, hospitalisations, and emergency activations increased worldwide. As part of the early pandemic response, several countries, including Spain, imposed nationwide lockdowns and restrictions to limit the spread of SARS-CoV-2. In addition, healthcare systems quickly adopted unprecedented measures to minimise disease transmission and prepare for the surge of COVID-19 patients. In line with recommendations by governments and international health organisations, consultations, routine diagnostic evaluations, and non-essential procedures were cancelled or deferred, causing disruptions to healthcare delivery and uptake worldwide with probable implications for healthcare quality and safety [1] and affecting the health outcomes of the most vulnerable populations [2].

During the first wave of the pandemic, various studies reported a decrease in emergency department (ED) visits, hospital admissions related to non-COVID conditions [3,4,5,6,7], and consultations in primary care [8]. Non-COVID respiratory diseases and cardiovascular emergencies seem to be the most affected conditions described to date [4,5,9,10,11], but there have also been reports of reductions in cerebrovascular and traumatology admissions [5,12]. In those cases, optimal emergency care is of the utmost importance to attenuate a potentially fatal disease course. In addition, medical care delay or avoidance due to the COVID-19 pandemic might have increased the morbidity and mortality risk associated with treatable conditions and may have contributed to the reported excess deaths directly or indirectly related to COVID-19 [13], as previously described [11].

Although health service disruptions decreased over time, the dynamics of the pandemic in each country implied that some interruptions persisted for more than one year after the onset [1]. The indirect consequences of the pandemic on healthcare for other diseases, known as collateral damage, has been scarcely quantified, especially after the first COVID-19 wave. In addition, there are few data on the impact of the pandemic on healthcare quality and patient safety indicators at the hospital level [2,14]. Here, we sought to evaluate changes in rates of non-COVID hospitalisations during the first year of the pandemic, as well as the impact of the pandemic on complications, in-hospital mortality, and readmissions among patients with non-COVID diseases in a university hospital in Spain. As a secondary objective, we aimed to identify the conditions with the greatest difference in healthcare quality indicators before and after the COVID-19 surge.

## 2. Materials and Methods

### 2.1. Setting and Study Population

We conducted a retrospective observational study of hospital admissions in a university hospital in Barcelona (Spain) from 1 March 2018 to 28 February 2021. Hospital del Mar is one of the four public reference hospitals in the city of Barcelona and attends medium- and high-complexity diseases in a catchment area of more than 300,000 inhabitants. The hospital has more than 400 conventional beds and 12 operating theatres. More than 95,000 patients are assessed annually in the ED.

The city of Barcelona is divided into four health areas based on territorial criteria, with integrated healthcare delivery and strong coordination mechanisms between primary and specialized care. Each area has a reference public hospital to attend to the population of the area. During the pandemic, sectorization criteria was maintained, both for COVID and non-COVID patients, except for paediatric and childbirth care.

We included all hospitalisations admitted through the ED. We excluded: (1) patients hospitalised for COVID-19 using discharge diagnostic codes based on the International Classification of Diseases, Tenth Revision (ICD-10) (B34.2, B97.29 and U07.1); (2) patients hospitalised with a diagnosis of COVID-19 suspicion (ICD-10 diagnostic codes Z20.828 and Z03.818); (3) episodes related to pregnancy and paediatric patients (<18 years) since they were referred to other centres from March to May 2020; and (4) all planned surgical and medical admissions because all planned activities were reduced in periods of increased COVID-19 admissions.

The study was approved by the Ethics Committee of Parc de Salut MAR (code number: 2021/9781), and no informed consent was required.

### 2.2. Pandemic Dynamics and Study Periods

In Spain, the first COVID-19 case was diagnosed on 31 January in the Canary Islands. The first case in Barcelona was confirmed on 25 February and the first hospital admission for COVID-19 at Hospital del Mar occurred on 1 March 2020. Thus, we defined the COVID period as being from 1 March 2020 to 28 February 2021 and divided it in three subperiods.

Containment measures in Spain started on 15 March, when the national lockdown became effective, and all residents were mandated to remain at home, and all non-essential businesses were temporarily closed. A gradual reopening started on 2 May 2020 until 21 June 2020. During the first national lockdown, face-to-face visits in both primary care and specialised care were cancelled to avoid infections and were redirected to telemedicine [15]. In our centre, elective surgeries were cancelled on 16 March, and the workforce turned to attending to the increasing number of COVID-19 patients and urgent non-COVID diseases. This situation implied the modification of conventional hospital circuits and the adaptation of professionals to take on new roles to respond to the emerging situation. We defined the complete lockdown period as 1 March 2020 to 21 June 2020. Although the complete lockdown in Spain started on 15 March, our hospital detected the first COVID-19 case on 28 February, and the crisis committee first met on 29 February, deciding the first containment measures on non-urgent activity.

From 22 June to October 2020, lockdown restrictions were gradually relaxed. In parallel with the drop in COVID-19 patients hospitalised in general wards and intensive care units, face-to-face activity was partially resumed as were elective surgical interventions. Thus, we defined the de-escalation period as 22 June to 10 October 2020.

National containment measures were applied again in Spain between 9 October 2020 and 9 May 2021. Partial lockdowns, mainly affecting non-essential activity and travel restrictions, were declared in the region of Barcelona on 25 October. On 27 December 2020, the first vaccines were administered to the Spanish population and cases gradually dropped, especially among the elderly, from the end of January onwards [16]. Therefore, we defined the partial lockdown period as 11 October to 28 February 2021.

Finally, as baseline, we defined the pre-COVID period as 1 March 2018 to 29 February 2020. To reduce bias due to interannual variability, we included data on 24 months of pre-COVID admissions.

### 2.3. Data Sources and Study Variables

Data were obtained from the minimum data set, which collects patient information and data on the principal diagnosis, and up to 14 comorbidities per patient, coded according to the International Classification of Diseases 10th Edition. Episodes were grouped using the APR-DRG (All Patient Refined Diagnosis Related Groups) version 36 and data were obtained on MDC (Major Diagnostic Category), DRG, and severity classification (from 1 to 4). In-hospital mortality was obtained from the discharge status.

Complications were defined as potentially avoidable and probably iatrogenic adverse events and were calculated based on the secondary diagnosis and based mainly on the patient safety indicators of the Agency for Healthcare Research and Quality (AHRQ) (https://www.qualityindicators.ahrq.gov/, accessed on 8 February 2022). Readmissions were defined as episodes with urgent admission within 30 days of discharge from an earlier clinically related hospitalisation and could be due to disease recurrence, worsening of a chronic condition, or complications from the previous episode. In-hospital mortality was defined as death during admission. Severity levels were obtained from the APR-DRG classification, consisting of values between 1 and 4, with one being the least severe category. Severity comparisons are allowed within the same APR-DRG only.

The main study variables were the weekly number of admissions (calculated through the mean number of admissions per natural week), complication rate (percentage of complications among hospitalised patients), in-hospital mortality rate (percentage of deaths among hospitalised patients), readmission rate (percentage of readmissions among hospitalised patients), and severity rate (percentage of episodes with maximum severity or 4). Explanatory variables included patient information, such as age, sex, Charlson Comorbidity Index (calculated through the percentage of 0, 1, 2, or 3 or more comorbidities), and information on diagnoses categorised using the MDC and APR-DRG.

### 2.4. Statistical Analysis

Patient and clinical characteristics were compared between periods by comparing absolute numbers and percentages. Statistically significant differences between periods were tested using the chi-square test for categorical variables, while the age distribution was checked for normality and was considered appropriate to apply the *t*-test.

We calculated the weekly number of hospitalisations during the COVID and pre-COVID periods. Weekly numbers across COVID subperiods were compared with the same weeks during the pre-COVID period to adjust for seasonality in hospital admissions. Rate ratios (RaR) and their 95% confidence intervals (95% CI) were calculated for comparison between periods based on the mean weekly rate. Risk ratios (RR) were calculated to compare the rates of complications, in-hospital mortality, readmissions, and severity. The Wald CI was calculated with 95% confidence. Data management and statistical analyses were performed using the SPSS Statistics programme, version 25 (IBM, Armonk, NY, USA). Statistical significance was set at *p* < 0.05.

## 3. Results

We retrospectively analysed 40,526 ED hospitalisations for non-COVID conditions from 1 March 2018 to 18 February 2021. Of these, 28,622 corresponded to the pre-COVID period and 11,904 to the COVID period. The baseline characteristics of the study population are shown in Table 1. The mean age and sex distribution of hospitalised patients were similar in the two periods (66.93 and 66.40 years; *p* = 0.099; 55.1% and 55.3% of men; *p* = 0.130, respectively). Hospitalisations during the COVID period showed a different distribution of the Charlson Comorbidity Index (*p* = 0.030) and source of hospital admission (*p* < 0.001), suggesting a higher percentage of patients without comorbidities (41.6% vs. 43.0% in pre-COVID and COVID period, respectively) and lower percentage of patients referred from primary care (6.4% vs. 5.3%, respectively).

**Figure 1:**
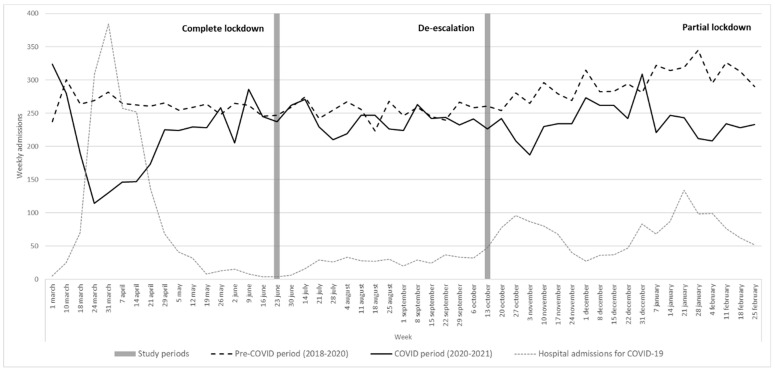
Weekly hospital admissions during the pre-COVID period (March 2018–February 2020) and COVID period (March 2020–February 2021). Weekly admissions of COVID-19 cases are displayed for comparison.

Weekly hospital admissions during the study period are shown in Figure 1. The weekly number of admissions was almost stable during the pre-COVID period, with a slightly increasing trend from October to March (flu period). During the COVID period, non-COVID admissions dropped sharply throughout the lockdown period and rose during the de-escalation but dropped again during the partial lockdown. The fall in non-COVID hospitalisations coincided with the peaks in COVID-19 hospitalisations during the study period.

A comparison of non-COVID-19 hospitalisations during the study period by MDC is shown in Table 2. The weekly admission rate dropped by 16.8% during the COVID period from 280.61 to 233.41 weekly admissions, reaching the lowest rate throughout the lockdown (199.59; −20.8% reduction from the previous period). We observed a significant reduction of admissions related to diseases of the respiratory system (RaR = 0.66, 95% CI: 0.63;0.70), diseases of the musculoskeletal system (RaR = 0.78, 95% CI: 0.72;0.84), and diseases of the circulatory system (RaR = 0.85, 95% CI: 0.81;0.90), among others. However, admissions increased for endocrine, nutritional, and metabolic diseases and diseases of the blood, especially from the de-escalation period onwards (RaR = 1.18 (1;1.38) and RaR = 1.18 (1.01;1.37), respectively).

The main quality indicators between study periods by MDC are compared in Table 3. Overall, the complication rate increased (from 1.84% to 2.23%, RR = 1.21, 95% CI: 1.05;1.4) and the readmissions rate decreased (from 4.21% to 3.21%, RR = 0.76, 95% CI: 0.68;0.85), whereas in-hospital mortality rates remained stable during the COVID period (from 4.1% to 4.45%, RR = 1.09, 95% CI: 0.98;1.2). However, some differences emerged when we considered MDC. Complications significantly increased for diseases of the musculoskeletal system (RR = 1.66, 95% CI: 1.09;2.51) as did hospital mortality for diseases of the digestive system (RR = 1.45, 95% CI: 1.07;1.96) or skin and subcutaneous tissue and breast diseases (RR = 2.44, 95% CI: 1.08;5.49), among others.

The RR for admissions, complications, mortality, readmissions, and severity by a selection of DRG of chronic and acute conditions are shown in Table 4. We observed a significant decrease in admissions for acute conditions, such as stroke and precerebral occlusions with infarction, acute myocardial infarction, and acute bronchitis, along with an increase in admissions for chronic conditions, such as anaemia and haematological and haematopoietic organ disorders. Despite the small number of events, there was no worsening of quality indicators, such as complications, in-hospital mortality, or readmissions, but we there was a general increase in severity, especially for those patients admitted for heart failure, other pneumonias, or acute bronchitis. Detailed figures for admissions, complications, mortality, readmissions, and severity by all DRG are available in the Supplementary Material.

## 4. Discussion

In this study, we evaluated the impact of the COVID-19 pandemic on hospitalisations for non-COVID conditions and on healthcare quality performance indicators in a university hospital in Spain. The number of non-COVID hospitalisations dramatically decreased, and this change persisted for up to a year after the onset of the pandemic. Hospitalisations related to diseases of the respiratory system, musculoskeletal system, and circulatory system also decreased, but admissions for endocrine, nutritional, and metabolic diseases and diseases of the blood increased, especially from the de-escalation period onwards. The disruption caused by the COVID-19 pandemic had a moderate impact on quality indicators in the hospital setting; the complication rate increased, whereas in-hospital mortality was similar in the two periods. The non-worsening of mortality rates is relevant since a general increase in severity was reported in most diseases during the COVID period.

Throughout the first COVID year, non-COVID hospitalisations decreased by 16.8%, with reductions of more than −20% during lockdown periods and of −5.9% during the de-escalation period. These figures are less pronounced than those reported in other countries during the first COVID wave [4,5,6,12]. The reasons for the decrease in Spain are probably multifactorial, stemming from a combination of community reluctance to seek healthcare for fear of infection [1] and adherence to stay-at-home orders. In addition, the reduction in visits and follow-up activity for chronic diseases in primary care [8,15] and outpatient activity may also have contributed to the decrease in the number of referrals [17,18]. Finally, some authors have hypothesised the existence of an increased threshold for referral and for hospitalization [19] as a potential contributor to the fall in ED hospital admissions.

There was a clear drop in hospitalisations for diseases of the respiratory system, such as chronic obstructive pulmonary disease exacerbations, acute bronchitis, and other pneumonias during the study period, including the de-escalation period. These results are consistent with those reported in other countries [4,5,6], mainly focused on the first COVID wave. Although we cannot rule out that patients with respiratory symptoms were misdiagnosed with COVID-19 during the first few weeks of the pandemic, hospitalisations for respiratory diseases remained lower across study periods and during the first year of the pandemic when all hospitalised patients were properly tested for COVID-19. Therefore, our data reflect a true decline in these hospitalisations, which may be explained by a combination effect of population-wide measures, such as mask-wearing and social distancing, as well as avoidance or delay in seeking care, especially among persons at increased risk for severe COVID-19 [20]. In addition, some studies reported improvements in medication adherence among patients with asthma and COPD during the COVID-19 pandemic, which may have a positive effect on improving disease control and reducing the number of exacerbations [21]. It has also been hypothesized that the decrease in air pollution during lockdown periods may potentially reduce respiratory disease exacerbations and short-term premature mortality [22,23]. In our study, severity increased among patients hospitalised for respiratory diseases, such as other pneumonias and acute bronchitis, pointing to a potential delay in care.

Previous studies also found a drop in admissions for acute cardiovascular and nervous system diseases during the initial COVID peak [4,5,6,7,8,9,24]. The decrease was observed especially during the complete and partial lockdown periods, whereas these indicators increased during the de-escalation period. Although the drop in hospitalisations may be partly explained by avoidance of medical care, another important factor was healthcare disruptions. During the lockdown periods, face-to-face primary care activity in Catalonia was drastically reduced, being partially switched towards telemedicine [15]. In addition, declines in ambulatory cardiovascular visits [8,25] (blood pressure monitoring in hypertensives and LDL control in ischaemic heart disease and stroke), as well as deferrals in less urgent cases, could all have led to an overall reduction in admissions. We found that admissions for myocardial infarction were reduced by 17% and those for stroke by 23%, accompanied by an increase in the severity of both diseases. As reported in other contexts, the reduction in admissions for nervous system diseases was accompanied by a marked drop in activations of stroke codes, and consequently, the number of recanalisation treatments (rtPA and thrombectomy) was greatly reduced during the pandemic [26,27]. In line with some studies [28], but contrasting with others [10], this study did not find higher in-hospital mortality for these conditions despite the greater severity of admitted patients, highlighting the maintenance of quality standards during the pandemic. Nevertheless, the drop in hospitalizations coincides with an increase in out-of-hospital cardiac arrest events [29,30] and greater mortality from cardiovascular diseases during the COVID period [4,31,32] observed in Spain and other countries.

Admissions for endocrine, nutritional, and metabolic diseases clearly increased during the de-escalation period with a non-significant trend to a higher complication rate and in-hospital mortality. Indeed, patients hospitalised for diabetes showed greater severity than those from the previous period. These trends may be influenced by the decrease in follow-up and monitoring activity from primary care in Catalonia (HbA1c control in type two diabetes mellitus, diabetic foot screening, and blood pressure control) from March to April 2020 [8,15], together with a worsening of disease control during the lockdown period due to poorer eating patterns and more sedentary behaviours [33]. Nevertheless, the admission rate tended to stabilise during the partial lockdown when primary care activity was partially recovered, combining both face-to-face and virtual visits [15]. Diabetes was one of the chronic conditions most strongly impacted by the reduction in healthcare activity due to COVID-19 followed by chronic obstructive pulmonary disease and hypertension [34,35]. These data indicate the need to adapt healthcare to monitor chronic conditions and provide care despite the pandemic in order to prevent treatment delays and avoidable complications. Given the existence of social inequalities in healthcare utilisation, it is likely that the results of this study would be worse among the most disadvantaged population.

The comparison of the main healthcare quality indicators between the COVID and pre-COVID periods revealed an overall increase in the complication rate but not in-hospital mortality rates. This information provides an overview of the impact of the pandemic on the quality of care for non-COVID diseases, thus far only shown specifically by some diseases [27,28]. The rise in complication rates may be explained by a mixture of changes in internal processes, high staff turnover, including among resident physicians, and healthcare reorganisation, which can be added to the effect of attending to patients with more severe diseases and greater management difficulties. In addition, the overcrowding in emergency departments may have also contributed to longer waiting times for hospitalisation as all patients had to be triaged before being hospitalised in a non-COVID unit [36]. To provide useful information at the hospital level, promote specific actions for improvement, and fine tune current contingency plans, there is a need for more in-depth analysis of patient groups identified as showing an increase in complications (i.e., musculoskeletal system diseases) or greater in-hospital mortality (i.e., diseases of the digestive system, kidney and urinary tract diseases, or subcutaneous skin and breast diseases). Nevertheless, the pandemic has highlighted some limitations of current quality indicators, especially those requiring human intervention in capturing quality data [14]. As proposed by Austin et al., data capture systems allowing data to be collected from hospitals without increasing clinician burden should be prioritised to provide timely information to rapidly evaluate care delivery.

The current study has some limitations. First, we present data from a single centre, which could introduce a selection bias if non-COVID patients from the reference area were admitted to other hospitals. Although we cannot rule out that a number of non-COVID-19 patients were hospitalised in other centers, the coordination mechanisms ensured that the criterion for sectorization was maintained across the study period both for COVID and non-COVID patient care. Second, our data was based on diagnostic codes. We used a standard approach for classifying diagnoses (ICD-10), but we cannot exclude some misclassification, especially during the first months of the pandemic when some respiratory diagnoses may have been classified as COVID-19 cases. However, we carried out a sensitivity analysis, including admissions with suspicion of COVID-19 (but not confirmed), and obtained similar results. Third, the non-worsening in-hospital mortality rate provides a positive overview of the effect of the pandemic within the hospital. However, these data do not show the impact on those patients not reaching the hospital facilities or reaching the emergency department in a terminal or irreversible condition, which may have resulted in higher mortality outside hospital care. Finally, the impact on readmissions, although apparently positive, should be interpreted with caution, as for some diseases, referral to other centres was modified by hospital occupancy criteria.

## 5. Conclusions

During the lockdown periods, the number of hospitalisations substantially decreased, while the severity of patients admitted for acute events increased, probably explained by a mixture of patient avoidance of emergency care for fear of COVID-19 [1], fewer primary care referrals, and a higher threshold for hospitalization [19]. This data reinforces findings reported in other countries [4,5,6]. After the peak of the pandemic, hospitalisations for metabolic and blood diseases increased, as did severity in general, which may be related to less strict control and less follow-up activity in primary care during the first months of the pandemic [18]. Hospital efforts to maintain quality and safety standards despite hospital disruptions translated into a moderate impact on healthcare quality indicators, with a significant increase in the complication rate but not in in-hospital mortality. Reduced hospitalisations for conditions requiring timely treatment may have significant public health consequences.

## Figures and Tables

**Table 1:** Baseline sociodemographic and clinical characteristics of non-COVID patients and comparison between COVID and Pre-COVID periods. Pre-COVID-19 period: March 2018 to February 2020; COVID-19: March 2020 to February 2021. SD: standard deviation. * *p*-values express the presence of statistically significant differences between the two periods (*t*-test for continuous variables, chi-square test for categorical).

	Pre-COVID-19 Period *N* (%)	COVID-19 Period *N* (%)	*p* Value *
Overall episodes	28,622	11,904	
Age, mean (SD)	66.93 (19.70)	66.40 (20.07)	0.099
Sex, *n* (%)			
Women, *n* (%)	12,857 (44.9)	5324 (44.7)	0.130
Men, *n* (%)	15,765 (55.1)	6580 (55.3)	
Charlson Comorbidity index, *n* (%)			
0	11,899 (41.6)	5108 (43.0)	0.030
1	5458 (19.1)	2255 (19.9)	
2	3873 (13.5)	1590 (13.4)	
≥3	7386 (25.8)	2923 (24.6)	
Source of hospital admission, *n* (%)			
Home	17,266 (60.4)	7572 (63.6)	<0.0001
Primary Care	1822 (6.4)	627 (5.3)	
Primary Care Emergency	632 (2.2)	448 (3.8)	
Nursing home	306 (1.1)	120 (1.0)	
Street	1786 (6.2)	358 (3.0)	
Other hospital	5199 (18.2)	2173 (18.3)	
Others	1558 (5.4)	599 (5.0)	
Unknown	26 (0.1)	7 (0.1)	

**Table 2:** Rate ratio of weekly number of admissions between pre-COVID and COVID periods by Major Diagnostic Categories (MDC). Weekly number of admissions pre-COVID-19 = number of unplanned emergency admissions/number of weeks pre-COVID period (2018–2020). Weekly number of admissions COVID-19 = number of unplanned emergency admissions/number of weeks COVID period (2020–2021). * *p* value < 0.05. ** Others category includes MDC with <100 episodes.

MDC	Pre-COVID-19 Period Number of Admissions (Weekly Rate)	COVID-19 Period Number of Admissions (Weekly Rate)	RaR (95% CI)	Complete Lockdown RaR (95% CI)	De-Escalation RaR (95% CI)	Partial Lockdown RaR (95% CI)
Total	28,622 (280.61)	11,904 (233.41)	0.83 (0.81;0.85) *	0.79 (0.76;0.82) *	0.94 (0.90;0.97) *	0.79 (0.76;0.82) *
Diseases and disorders of the respiratory system	5391 (52.85)	1791 (35.12)	0.66 (0.63;0.70) *	0.67 (0.61;0.73) *	0.87 (0.79;0.97) *	0.56 (0.51;0.61) *
Diseases and disorders of the circulatory system	4983 (48.85)	2128 (41.73)	0.85 (0.81;0.90) *	0.71 (0.64;0.78) *	0.95 (0.86;1.05)	0.90 (0.84;0.97) *
Diseases and disorders of the digestive system	3530 (34.61)	1431 (28.06)	0.81 (0.76;0.86) *	0.73 (0.65;0.82) *	0.88 (0.79;0.98) *	0.82 (0.74;0.90) *
Diseases and disorders of the nervous system	2251 (22.07)	952 (18.67)	0.85 (0.78;0.91) *	0.72 (0.62;0.84) *	0.97 (0.85;1.11)	0.84 (0.75;0.95) *
Diseases and disorders of the musculoskeletal system and connective tissue	2190 (21.47)	854 (16.75)	0.78 (0.72;0.84) *	0.79 (0.68;0.91) *	0.89 (0.77;1.02)	0.69 (0.61;0.79) *
Kidney and urinary tract diseases and disorders	2126 (20.84)	946 (18.55)	0.89 (0.82;0.96) *	0.87 (0.75;1.01)	0.91 (0.80;1.04)	0.89 (0.79;1.00)
Mental illnesses or disorders	1851 (18.15)	912 (17.88)	0.99 (0.91;1.07)	1.15 (1.01;1.32) *	0.93 (0.80;1.07)	0.90 (0.78;1.02)
Diseases and disorders of the hepatobiliary system and pancreas	1574 (15.43)	755 (14.8)	0.96 (0.88;1.05)	0.83 (0.70;0.98) *	1.08 (0.93;1.26)	0.97 (0.85;1.11)
Infectious and parasitic diseases (systemic or unspecified involvement)	847 (8.30)	345 (6.76)	0.81 (0.72;0.92) *	0.67 (0.53;0.86) *	0.95 (0.77;1.17)	0.81 (0.66;1.00)
Skin, subcutaneous tissue, and breast diseases and disorders	646 (6.33)	253 (4.96)	0.78 (0.68;0.91) *	0.94 (0.71;1.23)	0.93 (0.73;1.19)	0.58 (0.45;0.74) *
Diseases and disorders of the blood, the haematopoietic system, and the immune system	433 (4.25)	255 (5.00)	1.18 (1.01;1.37) *	0.85 (0.62;1.18)	1.08 (0.80;1.44)	1.47 (1.17;1.84) *
Endocrine, nutritional, and metabolic diseases and disorders	396 (3.88)	233 (4.57)	1.18 (1.00;1.38) *	1.27 (0.94;1.71)	1.37 (1.03;1.82) *	0.98 (0.75;1.28)
Diseases and disorders of the ear, nose, mouth, and throat	395 (3.87)	141 (2.76)	0.71 (0.59;0.87) *	1.54 (1.07;2.20) *	1.21 (0.85;1.73)	0.32 (0.23;0.45) *
Alcohol/drug use and alcohol/drug-induced organic mental disorders	352 (3.45)	135 (2.65)	0.77 (0.63;0.94) *	0.85 (0.61;1.18)	0.41 (0.27;0.61) *	1.15 (0.83;1.60)
Injuries, poisonings, and toxic effects of drugs	347 (3.40)	181 (3.55)	1.04 (0.87;1.25)	1.18 (0.83;1.67)	1.16 (0.87;1.56)	0.86 (0.63;1.16)
Diseases and disorders of the male reproductive system	278 (2.73)	159 (3.12)	1.14 (0.94;1.39)	0.94 (0.65;1.36)	1.22 (0.88;1.69)	1.26 (0.91;1.74)
Myeloproliferative diseases and disorders and poorly differentiated neoplasms	263 (2.58)	108 (2.12)	0.82 (0.66;1.03)	1.06 (0.73;1.55)	0.77 (0.49;1.20)	0.68 (0.48;0.98) *
Factors influencing health status and other contacts with health services	226 (2.22)	107 (2.10)	0.95 (0.75;1.19)	0.89 (0.60;1.34)	1.3 (0.88;1.93)	0.74 (0.49;1.10)
Others **	543 (5.32)	218 (4.27)	0.8 (0.69;0.94) *	0.72 (0.54;0.96) *	0.97 (0.73;1.28)	0.76 (0.59;0.97) *

**Table 3:** Comparison of complications, mortality, and readmissions between pre-COVID and COVID period by Major Diagnostic Categories (MDC). CI: confidence interval. RR: risk ratio. * *p* value < 0.05. ** Others category includes MDC with <100 episodes.

	Complications			In-Hospital Mortality			Readmissions		
MDC	Pre-COVID-19 Period *N* (%)	COVID-19 Period *N* (%)	RR (95% CI)	Pre-COVID-19 Period *N* (%)	COVID-19 Period *N* (%)	RR (95% CI)	Pre-COVID-19 Period *N* (%)	COVID-19 Period *N* (%)	RR (95% CI)
Total	600 (1.84%)	265 (2.23%)	1.21 (1.05;1.40) *	1337 (4.10%)	529 (4.45%)	1.09 (0.98;1.20)	1371 (4.21%)	381 (3.21%)	0.76 (0.68;0.85) *
Diseases and disorders of the respiratory system	58 (0.99%)	12 (0.67%)	0.67 (0.36;1.25)	384 (6.58%)	115 (6.42%)	0.98 (0.80;1.19)	517 (8.85%)	104 (5.81%)	0.66 (0.53;0.80) *
Diseases and disorders of the circulatory system	83 (1.49%)	45 (2.11%)	1.42 (0.99;2.04)	237 (4.25%)	79 (3.71%)	0.87 (0.68;1.12)	255 (4.57%)	79 (3.71%)	0.81 (0.63;1.04)
Diseases and disorders of the digestive system	119 (3.02%)	56 (3.91%)	1.30 (0.95;1.77)	116 (2.94%)	61 (4.26%)	1.45 (1.07;1.96) *	152 (3.85%)	27 (1.89%)	0.49 (0.33;0.73) *
Diseases and disorders of the nervous system	80 (3.49%)	39 (4.10%)	1.17 (0.81;1.71)	163 (7.11%)	74 (7.77%)	1.09 (0.84;1.42)	38 (1.66%)	11 (1.16%)	0.70 (0.36;1.36)
Diseases and disorders of the musculoskeletal system and connective tissue	60 (2.33%)	33 (3.86%)	1.66 (1.09;2.51) *	61 (2.37%)	28 (3.28%)	1.38 (0.89;2.15)	29 (1.13%)	16 (1.87%)	1.66 (0.91;3.04)
Kidney and urinary tract diseases and disorders	70 (2.72%)	18 (1.90%)	0.70 (0.42;1.17)	48 (1.86%)	31 (3.28%)	1.76 (1.13;2.74) *	107 (4.16%)	37 (3.91%)	0.94 (0.65;1.36)
Mental illnesses or disorders	1 (0.04%)	3 (0.33%)	7.35 (0.77;70.56)	4 (0.18%)	4 (0.44%)	2.45 (0.61;9.77)	11 (0.49%)	3 (0.33%)	0.67 (0.19;2.39)
Diseases and disorders of the hepatobiliary system and pancreas	29 (1.52%)	18 (2.38%)	1.57 (0.88;2.80)	85 (4.46%)	25 (3.31%)	0.74 (0.48;1.15)	98 (5.14%)	39 (5.17%)	1.00 (0.70;1.44)
Infectious and parasitic diseases (systemic or unspecified involvement)	25 (2.41%)	15 (4.35%)	1.80 (0.96;3.38)	92 (8.87%)	51 (14.78%)	1.67 (1.21;2.29) *	44 (4.24%)	24 (6.96%)	1.64 (1.01;2.66) *
Skin, subcutaneous tissue, and breast diseases and disorders	4 (0.50%)	4 (1.58%)	3.17 (0.8;12.58)	13 (1.62%)	10 (3.95%)	2.44 (1.08;5.49) *	28 (3.49%)	8 (3.16%)	0.91 (0.42;1.96)
Diseases and disorders of the blood, the haematopoietic system, and the immune system	6 (1.16%)	2 (0.78%)	0.68 (0.14;3.32)	6 (1.16%)	0 (0.00%)	-	11 (2.13%)	4 (1.57%)	0.74 (0.24;2.29)
Endocrine, nutritional, and metabolic diseases and disorders	4 (0.82%)	3 (1.29%)	1.57 (0.36;6.98)	7 (1.43%)	7 (3.00%)	2.10 (0.74;5.91)	15 (3.07%)	7 (3.00%)	0.98 (0.40;2.37)
Diseases and disorders of the ear, nose, mouth, and throat	5 (0.99%)	1 (0.71%)	0.71 (0.08;6.06)	13 (2.58%)	5 (3.55%)	1.37 (0.50;3.78)	17 (3.38%)	1 (0.71%)	0.21 (0.03;1.56)
Alcohol/drug use and alcohol/drug-induced organic mental disorders	1 (0.24%)	0 (0.00%)	-	0 (0.00%)	0 (0.00%)	-	1 (0.24%)	2 (1.48%)	6.09 (0.56;66.62)
Injuries, poisonings, and toxic effects of drugs	2 (0.47%)	3 (1.66%)	3.51 (0.59;20.85)	9 (2.12%)	6 (3.31%)	1.56 (0.56;4.32)	13 (3.07%)	5 (2.76%)	0.90 (0.33;2.49)
Diseases and disorders of the male reproductive system	5 (1.54%)	1 (0.63%)	0.41 (0.05;3.46)	8 (2.47%)	4 (2.52%)	1.02 (0.31;3.33)	16 (4.94%)	6 (3.77%)	0.76 (0.30;1.92)
Myeloproliferative diseases and disorders and poorly differentiated neoplasms	10 (3.27%)	2 (1.85%)	0.57 (0.13;2.55)	34 (11.11%)	17 (15.74%)	1.42 (0.83;2.43)	0 (0.00%)	0 (0.00%)	-
Factors influencing health status and other contacts with health services	3 (1.12%)	0 (0.00%)	-	8 (2.97%)	1 (0.93%)	0.31 (0.04;2.48)	4 (1.49%)	4 (3.74%)	2.51 (0.64;9.87)
Others **	35 (6.06%)	10 (5.26%)	0.87 (0.44;1.72)	49 (8.48%)	11 (5.79%)	0.68 (0.36;1.29)	15 (2.6%)	4 (2.11%)	0.81 (0.27;2.41)

**Table 4:** Comparison of admissions, complications, mortality, readmissions, and severity between pre-COVID and COVID periods by a selection of DRG for chronic and acute conditions. DRG: Diagnosis Related Group. RaR: rate ratio. RR: risk ratio. STEMI (ST elevation myocardial infarction) and NSTEMI (non-ST elevation myocardial infarction). * *p* value < 0.05.

DRG	AdmissionsRaR (95% CI)	ComplicationsRR (95% CI)	In-Hospital MortalityRR (95% CI)	ReadmissionsRR (95% CI)	SeverityRR (95% CI)
Stroke and precerebral occlusions with infarction	0.77 (0.66;0.90) *	2.36 (0.92;6.05)	1.12 (0.67;1.87)	1.14 (0.30;4.37)	1.09 (0.46;2.60)
Other pneumonia	0.70 (0.61;0.80) *	0.73 (0.16;3.43)	0.60 (0.34;1.08)	0.90 (0.48;1.70)	1.86 (1.34;2.60) *
Chronic obstructive pulmonary disease	0.55 (0.49;0.60) *	0.71 (0.25;2.07)	1.09 (0.68;1.76)	0.59 (0.44;0.79) *	1.20 (0.96;1.51)
Acute bronchitis and related symptoms	0.54 (0.44;0.65) *	-	0.69 (0.15;3.07)	1.26 (0.66;2.43)	1.98 (1.01;3.87) *
Acute myocardial infarction—STEMI and NSTEMI	0.66 (0.52;0.82) *	-	0.78 (0.27;2.27)	0.96 (0.32;2.87)	1.24 (0.50;3.12)
Heart failure	0.83 (0.76;0.91) *	0.38 (0.15;0.98) *	0.79 (0.56;1.11)	0.61 (0.42;0.89)	2.22 (1.69;2.92) *
Hip and femur fracture repair	0.80 (0.68;0.95) *	1.31 (0.70;2.45)	2.32 (1.04;5.17) *	0.63 (0.07;5.63)	1.64 (0.78;3.44)
Diabetes	1.16 (0.91;1.48)	3.52 (0.32;38.34)	0.59 (0.06;5.57)	0.25 (0.03;2.01)	2.20 (0.60;8.01)
Other anaemia and haematological and haematopoietic organ disorders	1.32 (1.11;1.57) *	0.51 (0.05;4.91)	-	0.77 (0.24;2.53)	0.77 (0.24;2.53)

## Data Availability

The data that support the findings of this study are available from the corresponding author, L.D., upon reasonable request.

## Supplementary Materials

The following supporting information can be downloaded at: https://www.mdpi.com/article/10.3390/jcm11071752/s1, Table S1: Comparison of admissions, complications, mortality, readmissions, and severity between pre-COVID and COVID period by Diagnosis Related Group (DRG).

## References

[1] World Health Organization COVID-19: Operational Guidance for Maintaining Essential Health Services During an Outbreak: Interim Guidance. 25 March 2020. https://apps.who.int/iris/handle/10665/331561.

[2] Braithwaite J. (2021). Quality of care in the COVID-19 era: A global perspective. IJQHC Commun..

[3] Jeffery M.M., D’Onofrio G., Paek H., Platts-Mills T.F., Soares W.E., Hoppe J.A., Genes N., Nath B., Melnick E.R. (2020). Trends in Emergency Department Visits and Hospital Admissions in Health Care Systems in 5 States in the First Months of the COVID-19 Pandemic in the US. JAMA Intern. Med..

[4] Santi L., Golinelli D., Tampieri A., Farina G., Greco M., Rosa S., Beleffi M., Biavati B., Campinoti F., Guerrini S. (2021). Non-COVID-19 patients in times of pandemic: Emergency department visits, hospitalizations and cause-specific mortality in Northern Italy. PLoS ONE.

[5] Baum A., Schwartz M.D. (2020). Admissions to Veterans Affairs Hospitals for Emergency Conditions during the COVID-19 Pandemic. JAMA.

[6] Bodilsen J., Nielsen P.B., Søgaard M., Dalager-Pedersen M., Speiser L.O.Z., Yndigegn T., Nielsen H., Larsen T.B., Skjøth F. (2021). Hospital admission and mortality rates for non-covid diseases in Denmark during covid-19 pandemic: Nationwide population based cohort study. BMJ.

[7] Katayama Y., Tanaka K., Kitamura T., Takeuchi T., Nakao S., Nitta M., Iwami T., Fujimi S., Uejima T., Miyamoto Y. (2021). Incidence and Mortality of Emergency Patients Transported by Emergency Medical Service Personnel during the Novel Corona Virus Pandemic in Osaka Prefecture, Japan: A Population-Based Study. J. Clin. Med..

[8] Coma E., Mora N., Méndez L., Benítez M., Hermosilla E., Fàbregas M., Fina F., Mercadé A., Flayeh S., Guiriguet C. (2020). Primary care in the time of COVID-19: Monitoring the effect of the pandemic and the lockdown measures on 34 quality of care indicators calculated for 288 primary care practices covering about 6 million people in Catalonia. BMC Prim. Care.

[9] Kiss P., Carcel C., Hockham C., Peters S.A.E. (2021). The impact of the COVID-19 pandemic on the care and management of patients with acute cardiovascular disease: A systematic review. Eur. Heart J. Qual. Care Clin. Outcomes.

[10] Bugger H., Gollmer J., Pregartner G., Wünsch G., Berghold A., Zirlik A., von Lewinski D. (2020). Complications and mortality of cardiovascular emergency admissions during COVID-19 associated restrictive measures. PLoS ONE.

[11] Nourazari S., Davis S.R., Granovsky R., Austin R., Straff D.J., Joseph J.W., Sanchez L.D. (2021). Decreased hospital admissions through emergency departments during the COVID-19 pandemic. Am. J. Emerg. Med..

[12] Mulholland R.H., Wood R., Stagg H.R., Fischbacher C., Villacampa J., Simpson C.R., Vasileiou E., McCowan C., Stock S.J., Docherty A.B. (2020). Impact of COVID-19 on accident and emergency attendances and emergency and planned hospital admissions in Scotland: An interrupted time-series analysis. J. R. Soc. Med..

[13] Kelly G., Petti S., Noah N. (2021). COVID-19, non-COVID-19 and excess mortality rates not comparable across countries. Epidemiol. Infect..

[14] Austin J.M., Kachalia A. (2020). The State of Health Care Quality Measurement in the Era of COVID-19: The Importance of Doing Better. JAMA.

[15] Lopez Segui F., Hernandez Guillamet G., Pifarré Arolas H., Marin-Gomez F.X., Ruiz Comellas A., Ramirez Morros A.M., Adroher Mas C., Vidal-Alaball J. (2021). Characterization and Identification of Variations in Types of Primary Care Visits before and during the COVID-19 Pandemic in Catalonia: Big Data Analysis Study. J. Med. Internet Res..

[16] Catalan Agency for Health Quality and Evaluation (AQuAS) Updated SARS-CoV-2 Data. https://aquas.gencat.cat/ca/actualitat/ultimes-dades-coronavirus/index.html#googtrans(ca|en).

[17] Birkmeyer J.D., Barnato A., Birkmeyer N., Bessler R., Skinner J. (2020). The Impact of The COVID-19 Pandemic on Hospital Admissions in the United States. Health Aff..

[18] Lopez-Villegas A., Bautista-Mesa R.J., Baena-Lopez M.A., Garzon-Miralles A., Castellano-Ortega M.A., Leal-Costa C., Peiro S. (2022). Impact of the COVID-19 Pandemic on Healthcare Activity in the Regional Hospitals of Andalusia (Spain). J. Clin. Med..

[19] Blecker S., Jones S.A., Petrilli C.M., Admon A.J., Weerahandi H., Francois F., Horwitz L.I. (2021). Hospitalizations for Chronic Disease and Acute Conditions in the Time of COVID-19. JAMA Intern. Med..

[20] Czeisler M.É., Marynak K., Clarke K.E.N., Salah Z., Shakya I., Thierry J.M., Ali N., McMillan H., Wiley J.F., Weaver M.D. (2020). Delay or Avoidance of Medical Care Because of COVID-19-Related Concerns—United States, June 2020. Morb. Mortal. Wkly. Rep..

[21] Kaye L., Theye B., Smeenk I., Gondalia R., Barrett M.A., Stempel D.A. (2020). Changes in medication adherence among patients with asthma and COPD during the COVID-19 pandemic. J. Allergy Clin. Immunol. Pract..

[22] Silva A.C.T., Branco P.T.B.S., Sousa S.I.V. (2022). Impact of COVID-19 Pandemic on Air Quality: A Systematic Review. Int. J. Environ. Res. Public Health.

[23] Schneider R., Masselot P., Vicedo-Cabrera A.M., Sera F., Blangiardo M., Forlani C., Douros J., Jorba O., Adani M., Kouznetsov R. (2022). Differential impact of government lockdown policies on reducing air pollution levels and related mortality in Europe. Sci. Rep..

[24] Romaguera R., Ribera A., Güell-Viaplana F., Tomás-Querol C., Muñoz-Camacho J.F., Agudelo V., en Representación de los Investigadores del Codi IAM (2020). Decrease in ST-segment elevation myocardial infarction admissions in Catalonia during the COVID-19 pandemic. Rev. Esp. Cardiol..

[25] Pifarré IArolas H., Vidal-Alaball J., Gil J., López F., Nicodemo C., Saez M. (2021). Missing Diagnoses during the COVID-19 Pandemic: A Year in Review. Int. J. Environ. Res. Public Health.

[26] Shatzkes D.R., Zlochower A.B., Steinklein J.M., Pramanik B.K., Filippi C.G., Azhar S., Wang J.J., Sanelli P.C. (2021). Impact of SARS-CoV-2 Pandemic on “Stroke Code” Imaging Utilization and Yield. Am. J. Neuroradiol..

[27] Rodrigues M., Grunho M., Rachão A., Silva E., Cordeiro A., Guilherme M., Pereira L. (2021). The impact of COVID-19 pandemic in stroke code activation and time from symptom onset to hospital arrival in a Portuguese comprehensive stroke centre. Rev. Neurol..

[28] Aktaa S., Yadegarfar M.E., Wu J., Rashid M., de Belder M., Deanfield J., Schiele F., Minchin M., Mamas M., Gale C.P. (2022). Quality of acute myocardial infarction care in England and Wales during the COVID-19 pandemic: Linked nationwide cohort study. BMJ Qual. Saf..

[29] Instituto Nacional de Estadística (INE) (2020). Defunciones Según la Causa de Muerte. https://www.ine.es/prensa/edcm_2020.pdf.

[30] Marijon E., Karam N., Jost D., Perrot D., Frattini B., Derkenne C., Sharifzadehgan A., Waldmann V., Beganton F., Narayanan K. (2020). Out-of-hospital cardiac arrest during the COVID-19 pandemic in Paris, France: A population-based, observational study. Lancet Public Health.

[31] Solomon M.D., Nguyen-Huynh M., Leong T.K., Alexander J., Rana J.S., Klingman J., Go A.S. (2021). Changes in Patterns of Hospital Visits for Acute Myocardial Infarction or Ischemic Stroke During COVID-19 Surges. JAMA.

[32] Baldi E., Sechi G.M., Mare C., Canevari F., Brancaglione A., Primi R., Klersy C., Palo A., Contri E., Ronchi V. (2020). Out-of-Hospital Cardiac Arrest during the Covid-19 Outbreak in Italy. N. Engl. J. Med..

[33] Sánchez E., Lecube A., Bellido D., Monereo S., Malagón M.M., Tinahones F.J., On Behalf of the Spanish Society for the Study of Obesity (2021). Leading Factors for Weight Gain during COVID-19 Lockdown in a Spanish Population: A Cross-Sectional Study. Nutrients.

[34] Chudasama Y.V., Gillies C.L., Zaccardi F., Coles B., Davies M.J., Seidu S., Khunti K. (2020). Impact of COVID-19 on routine care for chronic diseases: A global survey of views from healthcare professionals. Diabetes Metab. Syndr..

[35] Forde R., Arente L., Ausili D., De Backer K., Due-Christensen M., Epps A., Fitzpatrick A., Grixti M., Groen S., Halkoaho A. (2021). The impact of the COVID-19 pandemic on people with diabetes and diabetes services: A pan-European survey of diabetes specialist nurses undertaken by the Foundation of European Nurses in Diabetes survey consortium. Diabet. Med..

[36] Savioli G., Ceresa I.F., Gri N., Bavestrello Piccini G., Longhitano Y., Zanza C., Piccioni A., Esposito C., Ricevuti G., Bressan M.A. (2022). Emergency Department Overcrowding: Understanding the Factors to Find Corresponding Solutions. J. Pers. Med..

