# Supplementary material for: Impact of COVID-19 on Hospital Admissions and Healthcare Quality Indicators in Non-COVID Patients: A Retrospective Study of the First COVID-19 Year in a University Hospital in Spain
Source: J Clin Med. 2022 Mar 22;11(7):1752. doi: 10.3390/jcm11071752 (PMC8999691; doi:10.3390/jcm11071752)
Supplement: Supplementary file 1 [file jcm-11-01752-s001.zip › jcm-1610281-supplementary.pdf]

Table S1. Comparison of admissions, complications, mortality, readmissions and severity between pre-COVID and COVID period by Diagnosis Related Group (DRG)

MDC	GRD	Admissions RR (IC95%)	Complications RR (IC95%)	In-hospital mortality RR (IC95%)	Readmissions RR (IC95%)	Severity RR (IC95%)
Diseases and disorders of the nervous system	Craniotomy for trauma	0.94 (0.36;2.48)	1.08 (0.4;2.92)	0.43 (0.06;2.95)	-	4.33 (0.48;38.99)
Diseases and disorders of the nervous system	Viral meningitis	0.84 (0.6;1.17)	1.04 (0.58;1.88)	0.61 (0.26;1.39)	1.62 (0.28;9.39)	0.94 (0.42;2.12)
Diseases and disorders of the nervous system	Hypertensive encephalopathies	0.36 (0.11;1.23)	-	-	-	5.67 (0.47;68.03)
Diseases and disorders of the nervous system	Coma and stupor of non-traumatic origin	2.04 (0.66;6.32)	-	-	-	-
Diseases and disorders of the nervous system	Extracranial vascular procedures	0.92 (0.65;1.3)	1.25 (0.6;2.61)	0.74 (0.29;1.91)	2.22 (0.14;34.74)	0.81 (0.27;2.4)
Diseases and disorders of the nervous system	Other nervous system and related procedures	1.02 (0.41;2.53)	-	-	-	-
Diseases and disorders of the nervous system	Spinal disorders and injuries	0.58 (0.12;2.8)	-	-	-	-
Diseases and disorders of the nervous system	Neoplasms of the nervous system	1.18 (0.86;1.6)	-	0.69 (0.23;2.12)	-	0.35 (0.08;1.53)
Diseases and disorders of the nervous system	Degenerative disorders of the nervous system, except multiple sclerosis	1.31 (0.96;1.79)	-	1.24 (0.35;4.46)	-	2.07 (0.48;8.96)
Diseases and disorders of the nervous system	Multiple sclerosis and cerebellar ataxia	1.02 (0.31;3.39)	-	-	-	-
Diseases and disorders of the nervous system	Intracranial haemorrhage	0.77 (0.56;1.04)	-	1.12 (0.63;1.97)	2.66 (0.17;41.82)	0.94 (0.39;2.26)
Diseases and disorders of the nervous system	Stroke and precerebral occlusions with infarction	0.77 (0.66;0.9)	2.36 (0.92;6.05)	1.12 (0.67;1.87)	1.14 (0.3;4.37)	1.09 (0.46;2.6)
Diseases and disorders of the nervous system	Non-specific ACVA and precerebral occlusions without infarction	0.46 (0.23;0.92)	-	-	4.4 (0.3;64.53)	-
Diseases and disorders of the nervous system	Transient ischemic attack	0.65 (0.45;0.96)	-	-	-	-
Diseases and disorders of the nervous system	Cranial, peripheral and autonomic nerve disorders	1.1 (0.65;1.87)	-	-	-	-
Diseases and disorders of the nervous system	Bacterial and tuberculous infections of the nervous system	0.24 (0.07;0.78)	-	-	-	1.24 (0.22;6.92)
Diseases and disorders of the nervous system	Non-bacterial infections of the nervous system except viral meningitis	1.36 (0.69;2.67)	-	-	-	0.75 (0.07;7.51)

Diseases and disorders of the nervous system	Viral meningitis	0.29 (0.04;2.37)	-	-	-	-
Diseases and disorders of the nervous system	Coma and non-traumatic stupor	0.89 (0.5;1.57)	-	0.57 (0.07;4.76)	-	-
Diseases and disorders of the nervous system	Convulsions	0.68 (0.52;0.88)	0.86 (0.18;4.04)	6 (1.12;32.09)	1.5 (0.28;8.02)	2.4 (0.66;8.7)
Diseases and disorders of the nervous system	Migraine and other headaches	0.61 (0.32;1.17)	-	-	-	-
Diseases and disorders of the nervous system	Head injury with coma >1h or haemorrhage	0.82 (0.63;1.07)	4.95 (0.46;53.79)	2.17 (0.81;5.77)	0.49 (0.06;4.17)	0.55 (0.12;2.49)
Diseases and disorders of the nervous system	Cranial fracture and complicated intracranial injury, coma < 1h or no coma	0.51 (0.06;4.56)	-	-	-	-
Diseases and disorders of the nervous system	Concussion, uncomplicated intracranial injury, coma <1 hr or without coma	0.99 (0.76;1.3)	-	2.05 (0.42;9.93)	-	4.1 (0.38;44.55)
Diseases and disorders of the nervous system	Other diseases of the nervous system	1.36 (1.03;1.8)	-	1 (0.17;5.83)	-	0.75 (0.14;3.99)
Diseases and disorders of the nervous system	Tonsillectomy and/or adenoidectomy only, age >= 18,	0.82 (0.16;4.2)	-	1.67 (0.81;3.41)	-	2.5 (0.85;7.31)
Diseases and disorders of the eye	Lens procedures, except orbit	0.82 (0.26;2.6)	-	-	-	-
Diseases and disorders of the eye	Eye diseases, except major infections	0.45 (0.19;1.1)	-	-	-	-
Diseases and disorders of the ear, nose, mouth and throat	Major cranial/facial bone procedures	-	-	-	-	-
Diseases and disorders of the ear, nose, mouth and throat	Other major head and neck procedures	-	-	-	-	-
Diseases and disorders of the ear, nose, mouth and throat	Facial bone procedures except major cranial/facial bone proc, Major cranial/facial bone procedures	1.48 (0.6;3.69)	-	-	-	-
Diseases and disorders of the ear, nose, mouth and throat	Tonsillectomy and adenoidectomy	-	-	-	-	-
Diseases and disorders of the ear, nose, mouth and throat	Other ear, nose, mouth and throat procedures	0.73 (0.26;2.02)	-	-	-	0.93 (0.12;7.03)
Diseases and disorders of the ear, nose, mouth and throat	Head trauma with deep coma	-	-	-	-	-
Diseases and disorders of the ear, nose, mouth and throat	Ear, nose, mouth, throat and cranial/facial neoplasms	1.96 (1.15;3.36)	-	0.69 (0.22;2.18)	-	0.78 (0.19;3.15)
Diseases and disorders of the ear, nose, mouth and throat	Balance disorders	1.07 (0.62;1.84)	-	-	1.9 (0.13;28.8)	-
Diseases and disorders of the ear, nose, mouth and throat	Upper respiratory infections	0.33 (0.23;0.48)	-	-	-	0.87 (0.21;3.68)
Diseases and disorders of the ear, nose, mouth and throat	Dental diseases and disorders	1.63 (0.44;6.08)	-	-	-	-

Diseases and disorders of the ear, nose, mouth and throat	Other ear, nose, nose, mouth, throat and cranial/facial conditions	0.96 (0.66;1.4)	2.13 (0.14;33.12)	2.13 (0.14;33.12)	-	1.06 (0.1;11.38)
Diseases and disorders of the respiratory system	Major respiratory tract procedures	0.68 (0.07;6.53)	-	-	-	-
Diseases and disorders of the respiratory system	Other respiratory system procedures	0.43 (0.22;0.86)	-	0.78 (0.11;5.81)	2.35 (0.24;23.47)	1.04 (0.26;4.12)
Diseases and disorders of the respiratory system	Respiratory tract diseases with assisted ventilation for more than 96 hours	0.43 (0.21;0.88)	-	0.28 (0.04;1.85)	-	1.37 (0.34;5.52)
Diseases and disorders of the respiratory system	Respiratory failure	0.37 (0.26;0.53)	-	1.1 (0.45;2.68)	0.52 (0.17;1.59)	1.83 (0.63;5.37)
Diseases and disorders of the respiratory system	Pulmonary embolism	1.22 (0.96;1.56)	1.67 (0.11;26.36)	0.56 (0.15;2)	1 (0.24;4.1)	1.48 (0.59;3.72)
Diseases and disorders of the respiratory system	Major chest trauma	0.75 (0.55;1.04)	-	1.35 (0.13;14.6)	1.35 (0.13;14.6)	13.53 (1.62;113.04)
Diseases and disorders of the respiratory system	Respiratory neoplasms	0.94 (0.76;1.16)	0.72 (0.08;6.89)	0.96 (0.63;1.44)	-	1.18 (0.69;2.02)
Diseases and disorders of the respiratory system	Major pulmonary infections and inflammations	0.87 (0.75;1.01)	0.59 (0.07;5.23)	0.79 (0.54;1.17)	0.78 (0.43;1.44)	1.68 (1.25;2.28)
Diseases and disorders of the respiratory system	Bronchiolitis and respiratory syncytial virus pneumonia	0.17 (0.02;1.31)	-	-	-	-
Diseases and disorders of the respiratory system	Other pneumonia	0.7 (0.61;0.8)	0.73 (0.16;3.43)	0.6 (0.34;1.08)	0.9 (0.48;1.7)	1.86 (1.34;2.6)
Diseases and disorders of the respiratory system	Chronic obstructive pulmonary disease	0.55 (0.49;0.6)	0.71 (0.25;2.07)	1.09 (0.68;1.76)	0.59 (0.44;0.79)	1.2 (0.96;1.51)
Diseases and disorders of the respiratory system	Asthma	0.66 (0.5;0.85)	-	-	-	1.55 (0.55;4.4)
Diseases and disorders of the respiratory system	Interstitial and alveolar lung disease	1.1 (0.73;1.66)	-	0.41 (0.09;1.81)	0.51 (0.15;1.7)	1.39 (0.53;3.69)
Diseases and disorders of the respiratory system	Other respiratory diseases except minor signs, symptoms and diag, Minor signs, symptoms and diagnoses	0.92 (0.68;1.25)	1.1 (0.1;11.96)	0.74 (0.15;3.55)	0.47 (0.14;1.59)	1.23 (0.43;3.51)
Diseases and disorders of the respiratory system	Other minor diagnoses, signs and symptoms of the respiratory system	0.83 (0.68;1.02)	2.45 (0.15;38.8)	1.5 (0.64;3.54)	0.9 (0.39;2.09)	2.45 (0.72;8.3)
Diseases and disorders of the respiratory system	Acute bronchitis and related symptoms	0.54 (0.44;0.65)	-	0.69 (0.15;3.07)	1.26 (0.66;2.43)	1.98 (1.01;3.87)
Diseases and disorders of the circulatory system	Cardiac defibrillator and cardiac assist system implantation	0.65 (0.28;1.52)	1.57 (0.17;14.83)	-	-	-
Diseases and disorders of the circulatory system	Other cardiothoracic and thoracic vascular procedures	-	-	-	-	-
Diseases and disorders of the circulatory system	Heart valve procedures without iam or complex diagnosis	-	-	-	-	-

Diseases and disorders of the circulatory system	Other cardiothoracic and thoracic vascular procedures	1.41 (0.77;2.57)	-	-	1.44 (0.1;21.62)	-
Diseases and disorders of the circulatory system	Major abdominal vascular procedures	0.44 (0.13;1.52)	1.56 (0.24;10.28)	-	-	-
Diseases and disorders of the circulatory system	Implant, Permanent cardiac pacemaker without iam, heart failure or shock	1.02 (0.09;11.24)	-	-	2 (0.5;8)	-
Diseases and disorders of the circulatory system	Implant, Permanent cardiac pacemaker without iam, cardiac failure or shock	0.82 (0.63;1.07)	2.14 (0.74;6.16)	0.83 (0.09;7.87)	0.62 (0.14;2.87)	3.32 (0.76;14.51)
Diseases and disorders of the circulatory system	Percutaneous coronary interventions with iam	0.81 (0.69;0.95)	1.68 (0.6;4.65)	1.34 (0.58;3.12)	1.44 (0.61;3.38)	1.64 (0.87;3.08)
Diseases and disorders of the circulatory system	Percutaneous coronary interventions without iam	1.14 (0.96;1.35)	2.99 (1.33;6.71)	0.3 (0.04;2.47)	0.5 (0.19;1.32)	0.62 (0.3;1.3)
Diseases and disorders of the circulatory system	Pacemaker generator and cardiac defibrillator replacement	1.7 (0.52;5.57)	-	-	-	-
Diseases and disorders of the circulatory system	Revision of pacemaker and cardiac defibrillator except generator replacement	0.58 (0.12;2.8)	-	-	-	3.5 (0.36;34.33)
Diseases and disorders of the circulatory system	Other circulatory system procedures	0.71 (0.33;1.51)	-	0.72 (0.09;5.64)	-	-
Diseases and disorders of the circulatory system	Arterial procedures on lower extremity	0.82 (0.56;1.21)	0.99 (0.2;4.87)	0.41 (0.05;3.3)	1.24 (0.33;4.68)	1.06 (0.29;3.87)
Diseases and disorders of the circulatory system	Other peripheral vascular procedures	0.54 (0.3;0.95)	-	-	-	-
Diseases and disorders of the circulatory system	Acute myocardial infarction - iam	0.66 (0.52;0.82)	-	0.78 (0.27;2.27)	0.96 (0.32;2.87)	1.24 (0.5;3.12)
Diseases and disorders of the circulatory system	Cardiac catheterisation for coronary artery disease	0.9 (0.66;1.23)	-	2.26 (0.14;35.55)	0.45 (0.05;3.79)	-
Diseases and disorders of the circulatory system	Cardiac catheterisation for other non-coronary condition	0.79 (0.64;0.97)	0.86 (0.09;8.2)	0.52 (0.06;4.38)	0.81 (0.3;2.16)	1.55 (0.7;3.45)
Diseases and disorders of the circulatory system	Acute and subacute endocarditis	0.72 (0.36;1.44)	2.82 (0.45;17.66)	2.82 (0.66;11.95)	-	0.94 (0.31;2.85)
Diseases and disorders of the circulatory system	Heart failure	0.83 (0.76;0.91)	0.38 (0.15;0.98)	0.79 (0.56;1.11)	0.61 (0.42;0.89)	2.22 (1.69;2.92)
Diseases and disorders of the circulatory system	Cardiac arrest and shock	0.73 (0.4;1.35)	-	0.66 (0.31;1.42)	-	0.98 (0.49;1.98)
Diseases and disorders of the circulatory system	Peripheral vascular disorders and others	0.83 (0.6;1.16)	2.45 (0.16;38.38)	1.84 (0.67;5.02)	0.82 (0.09;7.66)	1.84 (0.43;7.91)
Diseases and disorders of the circulatory system	Coronary arteriosclerosis and angina pectoris	0.73 (0.54;0.98)	-	-	1.68 (0.41;6.81)	1.4 (0.13;15.14)
Diseases and disorders of the circulatory system	Hypertension	1.24 (0.89;1.73)	-	-	3.29 (0.3;35.41)	2.05 (0.58;7.33)
Diseases and disorders of the circulatory system	Trauma craniotomy	-	-	-	-	-

Diseases and disorders of the circulatory system	Congenital heart and valve diseases	0.98 (0.49;1.95)	-	2.08 (0.14;30.54)	-	-
Diseases and disorders of the circulatory system	Cardiac arrhythmias and conduction disorders	0.98 (0.81;1.18)	-	0.7 (0.14;3.41)	0.7 (0.23;2.13)	2.79 (0.63;12.3)
Diseases and disorders of the circulatory system	Thoracic pain	1.02 (0.71;1.46)	-	-	-	-
Diseases and disorders of the circulatory system	Syncope and collapse	1.31 (1;1.73)	-	1.55 (0.1;24.51)	1.55 (0.1;24.51)	-
Diseases and disorders of the circulatory system	Cardiomyopathy	2.04 (0.72;5.81)	-	-	-	-
Diseases and disorders of the circulatory system	Malfunction, reaction or complication of cardiovascular device or procedure, Cardiovascular,	0.71 (0.39;1.31)	-	-	-	5.71 (0.56;58.27)
Diseases and disorders of the circulatory system	Other circulatory system diagnoses	1.09 (0.77;1.54)	-	0.75 (0.15;3.73)	7.51 (0.86;65.37)	1.64 (0.63;4.26)
Diseases and disorders of the circulatory system	Craniotomy except for trauma	-	-	-	-	-
Diseases and disorders of the circulatory system	Ventricular shunt procedures	-	-	-	-	-
Diseases and disorders of the digestive system	Major procedures on stomach, oesophagus and duodenum	1.02 (0.57;1.83)	1.25 (0.48;3.24)	0.67 (0.07;5.94)	-	1.33 (0.68;2.63)
Diseases and disorders of the digestive system	Other procedures on stomach, oesophagus and duodenum	0.2 (0.05;0.87)	10 (0.94;105.93)	-	-	-
Diseases and disorders of the digestive system	Other small and large bowel procedures	0.85 (0.3;2.41)	-	-	-	-
Diseases and disorders of the digestive system	Peritoneal adhesiolysis	0.7 (0.36;1.35)	0.97 (0.11;8.48)	-	-	-
Diseases and disorders of the digestive system	Anal procedures	0.89 (0.59;1.33)	-	-	-	-
Diseases and disorders of the digestive system	Hernia procedures except inguinal, femoral and umbilical hernia procedures	0.76 (0.52;1.12)	1.01 (0.28;3.58)	2.69 (0.17;41.78)	-	2.69 (0.39;18.34)
Diseases and disorders of the digestive system	Inguinal, femoral and umbilical hernia procedures	0.81 (0.55;1.19)	0.84 (0.18;3.96)	-	0.84 (0.09;7.8)	-
Diseases and disorders of the digestive system	Other digestive tract surgical procedures	0.73 (0.4;1.35)	-	0.35 (0.05;2.54)	-	2.79 (0.95;8.19)
Diseases and disorders of the digestive system	Spinal procedures	-	-	-	-	-
Diseases and disorders of the digestive system	Major small bowel procedures	0.71 (0.49;1.02)	1.5 (0.91;2.46)	1.56 (0.67;3.6)	0.36 (0.05;2.79)	1.29 (0.84;1.99)
Diseases and disorders of the digestive system	Major large bowel procedures	0.87 (0.58;1.31)	1.04 (0.5;2.14)	-	-	0.51 (0.21;1.22)
Diseases and disorders of the digestive system	Gastric fundoplication	0.82 (0.16;4.2)	-	-	-	-

Diseases and disorders of the digestive system	Appendectomy with complex principal diagnosis	0.78 (0.55;1.1)	0.58 (0.13;2.59)	-	-	-
Diseases and disorders of the digestive system	Appendectomy without complex principal diagnosis	1.05 (0.85;1.28)	1.95 (0.28;13.69)	-	-	-
Diseases and disorders of the digestive system	Extracranial vascular procedures	-	-	-	-	-
Diseases and disorders of the digestive system	Digestive malignancy	0.85 (0.67;1.07)	2.06 (0.71;5.99)	1.6 (1.01;2.54)	-	1.2 (0.71;2.05)
Diseases and disorders of the digestive system	Peptic ulcer and gastritis	0.77 (0.59;1.02)	2.64 (0.17;41.59)	5.28 (0.49;57.25)	0.96 (0.32;2.91)	10.55 (1.2;92.75)
Diseases and disorders of the digestive system	Major diseases of the oesophagus	1.53 (0.78;2.99)	1.33 (0.09;19.64)	-	-	0.67 (0.07;6.68)
Diseases and disorders of the digestive system	Other esophageal diseases	0.5 (0.25;0.99)	-	4.1 (0.28;60.06)	-	8.2 (0.82;81.69)
Diseases and disorders of the digestive system	Diverticulitis and diverticulosis	0.9 (0.69;1.17)	-	-	0.65 (0.14;3.04)	-
Diseases and disorders of the digestive system	Inflammatory bowel disease	0.63 (0.41;0.96)	-	-	-	2.17 (0.38;12.32)
Diseases and disorders of the digestive system	Gastrointestinal vascular insufficiency	0.67 (0.46;0.99)	3.03 (0.19;47.16)	1.21 (0.41;3.62)	1.82 (0.46;7.22)	-
Diseases and disorders of the digestive system	Gastrointestinal obstruction	0.84 (0.67;1.07)	4.83 (0.44;52.64)	1.51 (0.51;4.5)	0.45 (0.13;1.52)	0.69 (0.15;3.26)
Diseases and disorders of the digestive system	Major bacterial infections of the digestive tract	1 (0.75;1.35)	-	0.81 (0.16;4.08)	0.12 (0.02;0.88)	1.27 (0.43;3.73)
Diseases and disorders of the digestive system	Other gastroenteritis, nausea and vomiting	0.77 (0.62;0.94)	2.66 (0.17;42.23)	1.33 (0.12;14.55)	0.76 (0.26;2.27)	0.27 (0.03;2.06)
Diseases and disorders of the digestive system	Abdominal pain	0.93 (0.75;1.17)	-	-	3.28 (0.56;19.36)	-
Diseases and disorders of the digestive system	Malfunction, reaction and complication of gastrointestinal device or proc, Gastrointestinal	0.8 (0.42;1.53)	-	-	2.54 (0.17;37.64)	1.27 (0.13;12.83)
Diseases and disorders of the digestive system	Other non-specific gastrointestinal bleeding	0.66 (0.52;0.84)	-	1.68 (0.64;4.41)	0.27 (0.06;1.11)	1.68 (0.64;4.41)
Diseases and disorders of the digestive system	Other digestive system diagnoses	0.85 (0.71;1.01)	0.8 (0.16;3.92)	1.5 (0.5;4.51)	0.16 (0.02;1.2)	0.92 (0.33;2.55)
Diseases and disorders of the digestive system	Other nervous system and related procedures	-	-	-	-	-
Diseases and disorders of the hepatobiliary system and pancreas	Major pancreas, liver and shunt procedures	1.19 (0.47;3.02)	1.14 (0.25;5.26)	-	-	1.14 (0.25;5.26)
Diseases and disorders of the hepatobiliary system and pancreas	Major biliary tract procedures	0.51 (0.17;1.52)	4 (0.79;20.32)	-	4 (0.31;51.03)	1.33 (0.18;9.66)

Diseases and disorders of the hepatobiliary system and pancreas	Cholecystectomy	0.87 (0.72;1.05)	1.25 (0.57;2.73)	1.57 (0.26;9.29)	0.94 (0.3;2.95)	1.45 (0.61;3.42)
Diseases and disorders of the hepatobiliary system and pancreas	Other hepatobiliary, pancreatic and abdominal procedures	0.82 (0.32;2.1)	-	-	-	0.71 (0.2;2.5)
Diseases and disorders of the hepatobiliary system and pancreas	Hepatic coma and other major liver disorders	1.06 (0.78;1.44)	-	0.38 (0.09;1.7)	1.28 (0.7;2.34)	0.48 (0.11;2.19)
Diseases and disorders of the hepatobiliary system and pancreas	Alcoholic liver disease	0.77 (0.54;1.1)	5.32 (0.5;57.08)	1.06 (0.21;5.27)	0.33 (0.04;2.58)	1.6 (0.4;6.38)
Diseases and disorders of the hepatobiliary system and pancreas	Malignant neoplasm of the hepatobiliary system or pancreas	0.83 (0.65;1.08)	-	0.56 (0.32;1)	-	0.61 (0.31;1.21)
Diseases and disorders of the hepatobiliary system and pancreas	Disorders of the pancreas except malignant neoplasm	0.99 (0.81;1.2)	1.03 (0.09;11.3)	1.55 (0.35;6.83)	0.99 (0.49;1.97)	2.06 (0.84;5.1)
Diseases and disorders of the hepatobiliary system and pancreas	Other liver disorders	0.53 (0.34;0.82)	-	-	-	-
Diseases and disorders of the hepatobiliary system and pancreas	Biliary tract and gallbladder disorders	1.38 (1.16;1.63)	-	1.11 (0.25;4.92)	0.41 (0.19;0.88)	1.65 (0.68;3.99)
Diseases and disorders of the hepatobiliary system and pancreas	Other intracranial percutaneous procedures	-	-	-	-	-
Diseases and disorders of the hepatobiliary system and pancreas	Percutaneous intracranial and extracranial vascular procedures	-	-	-	-	-
Diseases and disorders of the musculoskeletal system and connective tissue	Hip joint replacement	0.82 (0.64;1.04)	2.49 (0.96;6.44)	0.83 (0.27;2.51)	-	2.21 (0.88;5.56)
Diseases and disorders of the musculoskeletal system and connective tissue	Knee joint replacement	0.82 (0.39;1.7)	5 (0.51;49.16)	-	-	2.5 (0.17;36.21)
Diseases and disorders of the musculoskeletal system and connective tissue	Dorsal and lumbar scoliosis fusion procedures	-	-	-	-	-
Diseases and disorders of the musculoskeletal system and connective tissue	Dorsal and lumbar fusion procedures except for scoliosis	0.41 (0.16;1.07)	-	-	-	-

Diseases and disorders of the musculoskeletal system and connective tissue	Amputation of lower extremities except toes	1.16 (0.68;1.98)	0.88 (0.18;4.41)	1.76 (0.39;7.96)	-	0.7 (0.15;3.32)
Diseases and disorders of the musculoskeletal system and connective tissue	Hip and femur fracture repair	0.8 (0.68;0.95)	1.31 (0.7;2.45)	2.32 (1.04;5.17)	0.63 (0.07;5.63)	1.64 (0.78;3.44)
Diseases and disorders of the musculoskeletal system and connective tissue	Other significant hip and femur surgery	0.83 (0.41;1.67)	-	4.91 (0.49;48.76)	-	4.91 (1.05;23.03)
Diseases and disorders of the musculoskeletal system and connective tissue	Excision and decompression of intervertebral discs	1.22 (0.54;2.8)	-	-	-	-
Diseases and disorders of the musculoskeletal system and connective tissue	Skin graft diagnoses for musculoskeletal and connective tissue disorders, except hand	0.58 (0.12;2.8)	-	-	-	-
Diseases and disorders of the musculoskeletal system and connective tissue	Knee and lower leg procedures except foot	0.7 (0.55;0.88)	2.93 (0.42;20.49)	-	3.9 (0.89;17.12)	0.98 (0.1;9.27)
Diseases and disorders of the musculoskeletal system and connective tissue	Foot and toe procedures	0.79 (0.45;1.38)	-	-	-	2.59 (0.17;39.08)
Diseases and disorders of the musculoskeletal system and connective tissue	Shoulder, elbow and forearm procedures exc, Joint replacement	0.73 (0.51;1.04)	1.4 (0.13;15.06)	1.4 (0.13;15.06)	-	2.8 (0.18;43.82)
Diseases and disorders of the musculoskeletal system and connective tissue	Hand and wrist procedures	1.37 (0.93;2.02)	-	-	-	-
Diseases and disorders of the musculoskeletal system and connective tissue	Tendon, muscle and other soft tissue procedures	0.25 (0.1;0.63)	-	-	-	-
Diseases and disorders of the musculoskeletal system and connective tissue	Other musculoskeletal system and connective tissue procedures	0.5 (0.29;0.88)	-	-	-	-
Diseases and disorders of the musculoskeletal system and connective tissue	Cervical spinal fusion and other back/neck proc, Back/neck procedures except excision/decomp, Discal	-	-	-	-	-
Diseases and disorders of the musculoskeletal system and connective tissue	Shoulder and elbow replacement	2.04 (0.41;10.1)	-	-	-	-
Diseases and disorders of the musculoskeletal system and connective tissue	Femur fractures	0.57 (0.25;1.32)	-	-	-	-

Diseases and disorders of the musculoskeletal system and connective tissue	Pelvis fracture or hip dislocation	0.96 (0.58;1.57)	-	-	-	-
Diseases and disorders of the musculoskeletal system and connective tissue	Fracture or dislocation except femur, pelvis or back	0.91 (0.68;1.22)	-	-	2.23 (0.14;35.12)	-
Diseases and disorders of the musculoskeletal system and connective tissue	Pathological fractures and malignant musculoskeletal and connective tissue neoplasm	0.95 (0.6;1.5)	0.72 (0.08;6.57)	0.64 (0.19;2.15)	-	2.15 (0.58;7.95)
Diseases and disorders of the musculoskeletal system and connective tissue	Osteomyelitis, septic arthritis and other musculoskeletal infections	0.62 (0.3;1.25)	-	-	3.3 (0.23;48.13)	-
Diseases and disorders of the musculoskeletal system and connective tissue	Connective tissue disorders	1.1 (0.69;1.76)	1.85 (0.12;28.45)	0.46 (0.05;3.94)	3.7 (0.35;39.01)	0.21 (0.03;1.54)
Diseases and disorders of the musculoskeletal system and connective tissue	Other back and neck problems, fractures and injuries	0.57 (0.4;0.82)	-	1.79 (0.17;19.21)	3.58 (0.23;55.9)	-
Diseases and disorders of the musculoskeletal system and connective tissue	Malfunction, reaction or complication of an orthopaedic device or procedure, Orthopedic	0.4 (0.23;0.69)	-	-	1.71 (0.19;15.36)	-
Diseases and disorders of the musculoskeletal system and connective tissue	Other musculoskeletal system and connective tissue diagnoses	1.1 (0.84;1.46)	1.85 (0.12;29.11)	0.92 (0.09;10.02)	1.85 (0.47;7.18)	0.62 (0.07;5.82)
Skin, subcutaneous tissue and breast diseases and disorders	Skin grafting for skin and subcutaneous tissue diagnostics	0.37 (0.16;0.82)	-	-	2.79 (0.29;26.74)	2.79 (0.29;26.74)
Skin, subcutaneous tissue and breast diseases and disorders	Mastectomy procedures	-	-	-	-	-
Skin, subcutaneous tissue and breast diseases and disorders	Breast procedures except mastectomy	2.04 (0.81;5.14)	-	-	-	-
Skin, subcutaneous tissue and breast diseases and disorders	Other skin, subcutaneous tissue and related procedures	0.78 (0.45;1.34)	-	-	2.61 (0.17;39.56)	-
Skin, subcutaneous tissue and breast diseases and disorders	Skin ulcers	0.69 (0.47;1.02)	-	2.35 (0.67;8.26)	1.18 (0.24;5.79)	1.47 (0.28;7.67)
Skin, subcutaneous tissue and breast diseases and disorders	Major skin disorders	2.62 (0.98;7.04)	-	-	-	-
Skin, subcutaneous tissue and breast diseases and disorders	Malignant diseases of the breast	0.84 (0.35;2.02)	-	0.61 (0.08;4.52)	-	4.86 (0.52;45.32)
Skin, subcutaneous tissue and breast diseases and disorders	Cellulitis and other skin infections	0.74 (0.58;0.93)	0.92 (0.1;8.77)	8.31 (0.88;78.96)	0.58 (0.2;1.67)	1.58 (0.47;5.29)
Skin, subcutaneous tissue and breast diseases and disorders	Trauma to skin, subcutaneous tissue and open wounds	0.9 (0.65;1.26)	-	-	-	6.76 (0.72;63.48)

Skin, subcutaneous tissue and breast diseases and disorders	Other skin, subcutaneous tissue and breast diseases	1 (0.6;1.66)	-	-	-	-
Endocrine, nutritional and metabolic diseases and disorders	Tracheostomy with vm 96+ hours with extensive procedure	-	-	-	-	-
Endocrine, nutritional and metabolic diseases and disorders	Spinal disorders and injuries	-	-	-	-	-
Endocrine, nutritional and metabolic diseases and disorders	Adrenal gland procedures	-	-	-	-	-
Endocrine, nutritional and metabolic diseases and disorders	Surgical procedures for obesity	-	-	-	-	-
Endocrine, nutritional and metabolic diseases and disorders	Thyroid, parathyroid and thyroglossal tract procedures	0.51 (0.06;4.56)	-	-	-	-
Endocrine, nutritional and metabolic diseases and disorders	Other endocrine, nutritional and metabolic procedures	2.04 (0.29;14.48)	-	-	-	-
Endocrine, nutritional and metabolic diseases and disorders	Degenerative disorders of the nervous system, except multiple sclerosis	-	-	-	-	-
Endocrine, nutritional and metabolic diseases and disorders	Diabetes	1.16 (0.91;1.48)	3.52 (0.32;38.34)	0.59 (0.06;5.57)	0.25 (0.03;2.01)	2.2 (0.6;8.01)
Endocrine, nutritional and metabolic diseases and disorders	Malnutrition, failure to thrive and other nutritional diseases	1.36 (0.75;2.47)	-	-	-	-
Endocrine, nutritional and metabolic diseases and disorders	Hypovolaemia and related electrolyte disorders	1.22 (0.65;2.32)	-	3.33 (0.33;33.7)	-	-
Endocrine, nutritional and metabolic diseases and disorders	Congenital metabolic disorders	1.36 (0.23;8.14)	-	-	-	1.5 (0.18;12.46)
Endocrine, nutritional and metabolic diseases and disorders	Other endocrine disorders	0.65 (0.28;1.52)	-	-	-	3.14 (0.22;43.96)
Endocrine, nutritional and metabolic diseases and disorders	Other non-hypovolaemic electrolyte disorders	1.41 (0.97;2.06)	-	-	0.72 (0.07;7.73)	1.44 (0.09;22.5)

Endocrine, nutritional and metabolic diseases and disorders	Non-hypovolemic sodium disorders	1.2 (0.81;1.79)	-	-	0.68 (0.14;3.32)	-
Endocrine, nutritional and metabolic diseases and disorders	Thyroid disorders	1.22 (0.29;5.12)	-	-	-	-
Kidney and urinary tract diseases and disorders	Intracranial haemorrhage	-	-	-	-	-
Kidney and urinary tract diseases and disorders	Renal transplantation	0.64 (0.47;0.87)	0.95 (0.52;1.72)	-	1 (0.38;2.59)	2.65 (0.84;8.36)
Kidney and urinary tract diseases and disorders	Major bladder procedures	0.41 (0.05;3.49)	-	-	-	-
Kidney and urinary tract diseases and disorders	Kidney and urinary tract procedures for neoplasia	10.2 (1.19;87.28)	-	-	-	-
Kidney and urinary tract diseases and disorders	Kidney and urinary tract procedures for non-malignant processes	0.81 (0.6;1.1)	0.36 (0.05;2.85)	1.67 (0.29;9.76)	2.51 (0.36;17.4)	0.68 (0.2;2.37)
Kidney and urinary tract diseases and disorders	Repair of access device and vessels for renal dialysis	3.06 (0.51;18.31)	-	-	-	-
Kidney and urinary tract diseases and disorders	Other bladder procedures	1.43 (0.54;3.75)	-	-	-	1.43 (0.11;19.2)
Kidney and urinary tract diseases and disorders	Urethral and transurethral procedures	0.96 (0.52;1.77)	-	-	-	2.13 (0.14;31.84)
Kidney and urinary tract diseases and disorders	Other kidney and urinary tract and related procedures	-	-	-	-	-
Kidney and urinary tract diseases and disorders	Stroke and precerebral occlusions with infarction	-	-	-	-	-
Kidney and urinary tract diseases and disorders	Non-specific ACVA and precerebral occlusions without infarction	-	-	-	-	-
Kidney and urinary tract diseases and disorders	Neoplasms of the kidney and urinary tract	0.94 (0.57;1.54)	-	1.3 (0.54;3.16)	-	0.87 (0.18;4.15)
Kidney and urinary tract diseases and disorders	Nephritis and nephrosis	0.78 (0.28;2.2)	-	-	-	-
Kidney and urinary tract diseases and disorders	Kidney and urinary tract infections	0.95 (0.85;1.07)	0.31 (0.04;2.47)	1.57 (0.73;3.38)	0.65 (0.36;1.17)	1.51 (0.82;2.79)
Kidney and urinary tract diseases and disorders	Urinary calculi and acquired upper urinary tract obstruction	0.88 (0.63;1.24)	-	-	1.16 (0.22;6.1)	-
Kidney and urinary tract diseases and disorders	Malfunction, reaction or complication of genitourinary surgical devices	0.67 (0.38;1.17)	-	-	0.77 (0.18;3.24)	6.13 (0.59;63.16)
Kidney and urinary tract diseases and disorders	Other kidney and urinary tract diagnoses, signs and symptoms	0.96 (0.71;1.29)	-	2.13 (0.14;33.46)	0.64 (0.18;2.24)	-
Kidney and urinary tract diseases and disorders	Acute kidney damage	0.99 (0.82;1.2)	6.19 (0.65;59.06)	1.43 (0.62;3.27)	0.85 (0.36;2.01)	1.81 (0.67;4.89)

Kidney and urinary tract diseases and disorders	Chronic kidney disease	-	-	-	-	-
Kidney and urinary tract diseases and disorders	Cranial, peripheral and autonomic nerve disorders	0.72 (0.48;1.08)	-	-	2.84 (0.18;44.03)	-
Diseases and disorders of the male reproductive system	Major procedures on male pelvis	2.04 (0.29;14.48)	-	-	-	-
Diseases and disorders of the male reproductive system	Transurethral prostatectomy	-	-	-	-	-
Diseases and disorders of the male reproductive system	Penile, testicular and scrotal procedures	0.95 (0.49;1.83)	-	-	-	-
Diseases and disorders of the male reproductive system	Other surgical proc, Surgical procedures of male genital ap, Male genital surgery	1.22 (0.29;5.12)	1.67 (0.16;17.89)	-	-	3.33 (0.49;22.9)
Diseases and disorders of the male reproductive system	Bacterial and tuberculous infections of the nervous system	-	-	-	-	-
Diseases and disorders of the male reproductive system	Non-bacterial infections of the nervous system except viral meningitis	-	-	-	-	-
Diseases and disorders of the male reproductive system	Neoplasms of the male genital tract	1.59 (0.79;3.19)	-	0.96 (0.26;3.62)	-	1.29 (0.3;5.43)
Diseases and disorders of the male reproductive system	Diagnoses of male genital tract except neoplasm	1.18 (0.95;1.46)	-	0.87 (0.08;9.46)	0.94 (0.36;2.49)	1.73 (0.57;5.26)
Diseases and disorders of the female reproductive system	Pelvic evisceration, radical hysterectomy and other radical gynaecological procedures	-	-	-	-	-
Diseases and disorders of the female reproductive system	Proc, On the uterus and adnexa due to malignant ovarian or adnexal neoplasia	4.08 (0.37;44.98)	-	-	-	-
Diseases and disorders of the female reproductive system	Proc, On the uterus and adnexa for malignant neoplasm other than ovarian or adnexal malignancies	-	-	-	-	-
Diseases and disorders of the female reproductive system	Procedures on uterus and adnexa non-malignant process, except leiomyoma	1.31 (0.82;2.1)	-	-	-	-
Diseases and disorders of the female reproductive system	Proc, Reconstruction of the female genital tract	-	-	-	-	-
Diseases and disorders of the female reproductive system	Dilatation and curettage for non-obstetric diagnosis	-	-	-	-	-
Diseases and disorders of the female reproductive system	Other surgical procedures on female genital tract	0.64 (0.26;1.61)	-	-	-	-
Diseases and disorders of the female reproductive system	Procedures on uterus and appendages for leiomyoma	1.36 (0.23;8.14)	-	-	-	-
Diseases and disorders of the female reproductive system	Alteration of consciousness	-	-	-	-	-
Diseases and disorders of the female reproductive system	Convulsions	-	-	-	-	-
Diseases and disorders of the female reproductive system	Malignant disorders of the female reproductive system	1.55 (0.85;2.81)	-	0.66 (0.19;2.3)	-	1.05 (0.33;3.4)

Diseases and disorders of the female reproductive system	Infections of the female reproductive system	0.61 (0.35;1.05)	-	-	-	-
Diseases and disorders of the female reproductive system	Other menstrual and female reproductive system diseases	-	-	-	-	-
Diseases and disorders of the blood, the hematopoietic system and the immune system	Migraine and other headaches	-	-	-	-	-
Diseases and disorders of the blood, the hematopoietic system and the immune system	Head injury with coma >1h or haemorrhage	-	-	-	-	-
Diseases and disorders of the blood, the hematopoietic system and the immune system	Skull fracture and complicated intracranial injury, coma < 1h or without coma	-	-	-	-	-
Diseases and disorders of the blood, the hematopoietic system and the immune system	Other antepartum diagnoses	-	-	-	-	-
Diseases and disorders of the blood, the hematopoietic system and the immune system	Concussion, closed skull fracture neom, uncomplicated intracranial injury, coma <1 hr or without coma	-	-	-	-	-
Diseases and disorders of the blood, the hematopoietic system and the immune system	Other diseases of the nervous system	-	-	-	-	-
Diseases and disorders of the blood, the hematopoietic system and the immune system	Anoxia and other severe brain damage	-	-	-	-	-
Diseases and disorders of the blood, the hematopoietic system and the immune system	Splenectomy	-	-	-	-	-
Diseases and disorders of the blood, the hematopoietic system and the immune system	Other haematological and haematopoietic organ procedures	0.68 (0.14;3.37)	-	-	-	-
Diseases and disorders of the blood, the hematopoietic system and the immune system	Major haematological/immunological diag except sickle cell and coagulation crises	0.83 (0.52;1.33)	-	-	-	-
Diseases and disorders of the blood, the hematopoietic system and the immune system	Coagulation and platelet disorders	1.12 (0.54;2.34)	-	-	-	-
Diseases and disorders of the blood, the hematopoietic system and the immune system	Sickle cell disease crises	0.94 (0.36;2.48)	-	-	-	-

Diseases and disorders of the blood, the hematopoietic system and the immune system	Other anaemia and haematological and haematopoietic organ disorders	1.32 (1.11;1.57)	0.51 (0.05;4.91)	-	0.77 (0.24;2.53)	0.77 (0.24;2.53)
Myeloproliferative diseases and disorders and poorly differentiated neoplasms	Major surgical procedure for lymphatic/hematopoietic/other neoplasms	2.04 (0.13;32.6)	-	-	-	-
Myeloproliferative diseases and disorders and poorly differentiated neoplasms	Other surgical procedure for lymphatic/hematopoietic/other neoplasms	0.68 (0.33;1.39)	0.6 (0.08;4.54)	1 (0.12;8.56)	-	0.38 (0.05;2.64)
Myeloproliferative diseases and disorders and poorly differentiated neoplasms	Acute leukemia	1.4 (0.81;2.41)	-	1.09 (0.27;4.4)	-	1.82 (0.55;6.02)
Myeloproliferative diseases and disorders and poorly differentiated neoplasms	Lymphoma, myeloma and non-acute leukemia	0.66 (0.47;0.92)	-	1.62 (0.84;3.1)	-	1.76 (0.79;3.94)
Myeloproliferative diseases and disorders and poorly differentiated neoplasms	Radiotherapy	-	-	-	-	-
Myeloproliferative diseases and disorders and poorly differentiated neoplasms	Lymphoid and other neoplasms and neoplasms of questionable behaviour	1.08 (0.68;1.7)	-	0.63 (0.14;2.92)	-	2.21 (0.82;5.94)
Myeloproliferative diseases and disorders and poorly differentiated neoplasms	Chemotherapy for acute leukaemia	-	-	-	-	-
Myeloproliferative diseases and disorders and poorly differentiated neoplasms	Other chemotherapy	-	-	-	-	-
Infectious and parasitic diseases (systemic or unspecified involvement)	Infectious diseases and parasitosis including hiv with surgical procedure	0.94 (0.6;1.46)	1.09 (0.49;2.4)	2 (1.01;3.96)	2.18 (0.14;33.59)	1.12 (0.79;1.59)
Infectious and parasitic diseases (systemic or unspecified involvement)	Post-operative, post-traumatic and other device infections with surgical procedure	1.33 (0.66;2.66)	3.08 (0.31;30.59)	-	-	-
Infectious and parasitic diseases (systemic or unspecified involvement)	Septicaemia and disseminated infections	0.77 (0.64;0.94)	1.5 (0.45;5.06)	1.26 (0.89;1.79)	1.18 (0.55;2.54)	1.02 (0.77;1.35)
Infectious and parasitic diseases (systemic or unspecified involvement)	Post-operative, post-traumatic and other device infections	0.74 (0.49;1.11)	1.38 (0.13;14.65)	2.75 (0.18;42.68)	1.83 (0.32;10.48)	-
Infectious and parasitic diseases (systemic or unspecified involvement)	Fever	0.99 (0.73;1.35)	-	-	1.71 (0.54;5.37)	-

Infectious and parasitic diseases (systemic or unspecified involvement)	Viral disease	0.9 (0.49;1.65)	-	-	-	-
Infectious and parasitic diseases (systemic or unspecified involvement)	Other systemic infections and parasitosis	0.76 (0.55;1.05)	-	1.79 (0.31;10.38)	2.35 (0.9;6.13)	0.67 (0.2;2.28)
Mental illnesses or disorders	Eye and orbit procedures	-	-	-	-	-
Mental illnesses or disorders	Diagnosis of mental illness with surgical procedure	0.41 (0.05;3.49)	-	-	-	-
Mental illnesses or disorders	Schizophrenia	1.07 (0.92;1.25)	-	-	-	0.38 (0.08;1.73)
Mental illnesses or disorders	Major depressive disorders and other/unspecified psychoses	1.07 (0.9;1.27)	-	-	-	0.95 (0.29;3.13)
Mental illnesses or disorders	Personality and impulse control disorders	0.64 (0.43;0.95)	-	-	-	-
Mental illnesses or disorders	Bipolar disorders	0.92 (0.76;1.12)	-	-	-	0.95 (0.25;3.61)
Mental illnesses or disorders	Depression except major depressive disorder	0.79 (0.48;1.29)	-	-	-	-
Mental illnesses or disorders	Adjustment disorders and neuroses except depressive diagnoses	0.98 (0.66;1.46)	-	-	-	-
Mental illnesses or disorders	Acute anxiety states and delirium	1.12 (0.91;1.38)	1.81 (0.11;28.76)	2.72 (0.46;16.08)	0.26 (0.03;2.08)	1.81 (0.26;12.73)
Mental illnesses or disorders	Organic mental health disorders	1.09 (0.77;1.54)	-	0.94 (0.09;10.08)	0.63 (0.07;5.85)	-
Mental illnesses or disorders	Behavioural disorders	0.86 (0.43;1.75)	-	-	-	-
Mental illnesses or disorders	Eating disorders	1.53 (0.34;6.83)	-	-	-	-
Mental illnesses or disorders	Other mental health disorders	0.85 (0.53;1.35)	-	-	2.4 (0.16;36.88)	-
Alcohol/drug use and alcohol/drug-induced organic mental disorders	Drug and alcohol abuse or dependence, voluntary discharge	-	-	-	-	-
Alcohol/drug use and alcohol/drug-induced organic mental disorders	Drug and alcohol abuse or dependence with rehabilitation or combined rehab/detox treatment, Rehab/detoxification treatment	0.54 (0.33;0.88)	-	-	-	-
Alcohol/drug use and alcohol/drug-induced organic mental disorders	Opiate abuse or dependence	0.43 (0.23;0.83)	-	-	-	-
Alcohol/drug use and alcohol/drug-induced organic mental disorders	Cocaine abuse or dependence	0.55 (0.36;0.84)	-	-	-	-
Alcohol/drug use and alcohol/drug-induced organic mental disorders	Alcohol abuse or dependence	1.08 (0.75;1.54)	-	-	3.78 (0.35;40.62)	1.89 (0.28;12.99)

Alcohol/drug use and alcohol/drug-induced organic mental disorders	Other drug abuse or dependence	0.94 (0.53;1.66)	-	-	-	-
Injuries, poisonings and toxic effects of drugs	Extensive surgical procedure for other treatment complications	0.19 (0.02;1.44)	11 (1.7;71.28)	-	-	-
Injuries, poisonings and toxic effects of drugs	Moderately extensive surgical procedure for other treatment complications	0.71 (0.32;1.59)	-	-	-	2.88 (0.2;40.8)
Injuries, poisonings and toxic effects of drugs	Non-extensive surgical procedure for other treatment complications	0.45 (0.1;2.1)	-	-	-	-
Injuries, poisonings and toxic effects of drugs	Haemorrhage or haematoma due to complication	0.64 (0.34;1.23)	-	-	-	-
Injuries, poisonings and toxic effects of drugs	Allergic reactions	0.68 (0.27;1.71)	-	-	-	6 (0.65;55)
Injuries, poisonings and toxic effects of drugs	Poisoning by medicinal agents	1.18 (0.85;1.66)	-	1.15 (0.2;6.66)	0.57 (0.06;5.38)	2.07 (0.66;6.45)
Injuries, poisonings and toxic effects of drugs	Other complications of treatment	1.25 (0.79;1.97)	-	-	2.18 (0.52;9.07)	-
Injuries, poisonings and toxic effects of drugs	Other diagnoses of injury, poisoning and toxic effect	0.89 (0.5;1.57)	-	1.15 (0.23;5.68)	-	0.46 (0.06;3.64)
Injuries, poisonings and toxic effects of drugs	Toxic effects of non-medicinal substances	1.42 (0.83;2.42)	-	1.43 (0.09;21.79)	-	0.96 (0.17;5.28)
Injuries, poisonings and toxic effects of drugs	Overdosis	1.68 (1.02;2.77)	-	-	-	1.82 (0.33;10.15)
Injuries, poisonings and toxic effects of drugs	Infections and other eye diseases	-	-	-	-	-
Burns	Partial thickness burns without skin grafting	4.08 (0.37;44.98)	-	-	-	-
Factors influencing health status and other contacts with health services	Procedure with diag, Rehab, aftercare or other contact with health care services	0.56 (0.23;1.37)	-	-	-	-
Factors influencing health status and other contacts with health services	Rehabilitation	0.97 (0.58;1.64)	-	-	2.1 (0.14;31.89)	0.7 (0.08;6.32)
Factors influencing health status and other contacts with health services	Signs, symptoms and other factors affecting health status	1.17 (0.8;1.71)	-	-	-	-
Factors influencing health status and other contacts with health services	Other aftercare and convalescence	0.9 (0.62;1.32)	-	0.45 (0.05;3.74)	4.53 (0.42;48.42)	0.57 (0.07;4.9)
HIV infections	HIV with multiple major HIV-related conditions	0.65 (0.28;1.52)	-	-	-	2.24 (1.04;4.85)
HIV infections	HIV with major HIV-related conditions	0.5 (0.24;1.03)	-	-	-	-

HIV infections	HIV with multiple major significant HIV-related conditions	-	-	-	-	-
HIV infections	HIV with one significant HIV-related condition or no significant HIV-related condition	0.98 (0.49;1.95)	-	-	-	-
Major polytrauma	Other major head and neck procedures	-	-	-	-	-
Major polytrauma	Craniotomy for significant multiple trauma	-	-	-	-	-
Major polytrauma	Extensive abdominal/thoracic procedures for significant multiple trauma	1.36 (0.48;3.82)	3 (0.34;26.19)	-	-	0.75 (0.3;1.89)
Major polytrauma	Musculoskeletal and other procedures for significant multiple trauma	0.33 (0.11;0.94)	-	-	-	-
Major polytrauma	Facial bone procedures except major cranial/facial bone proc, Major cranial/facial bone procedures	-	-	-	-	-
Major polytrauma	Multiple significant trauma without surgical procedure	0.88 (0.51;1.51)	-	0.77 (0.09;6.96)	-	3.47 (0.63;19.14)
Unvalid category	Extensive procedure unrelated to principal diagnosis	0.48 (0.22;1.04)	1.7 (0.4;7.23)	-	1.06 (0.14;8.27)	-
Unvalid category	Moderately extensive procedure unrelated to principal diagnosis	0.38 (0.17;0.84)	-	-	1.36 (0.18;10.41)	1.55 (0.4;5.98)
Unvalid category	Non-extensive procedure unrelated to principal diagnosis	0.39 (0.15;1.02)	-	-	-	5.2 (0.94;28.76)
